# Photomechanical Azopolymers and Digital Polarization Optics: A Versatile Platform for Surface Microstructure Fabrication

**DOI:** 10.1021/acsaom.5c00038

**Published:** 2025-04-23

**Authors:** Jonas Strobelt, Svetlana Santer, Heba Abourahma, Mika Music, Zay Farzan, Phillip Nezamis, Ryan Leon, David J. McGee

**Affiliations:** † Universität Potsdam, Institut für Physik und Astronomie, Karl-Liebknecht-Str.24/25, 14476 Potsdam-Golm, Germany; ‡ The College of New Jersey, Department of Chemistry, 2000 Pennington Road, Ewing, New Jersey 08628, United States; § 3280 The College of New Jersey, Department of Physics, 2000 Pennington Road, Ewing, New Jersey 08628, United States; ∥ Berliner Hochschule für Technik, Fachbereich 2, Luxemburger Str. 10, 13353 Berlin, Germany

**Keywords:** azobenzene containing polymers, surface relief grating, spatial light modulator, maskless lithography, direct laser-writing, diffractive optics, holography, dynamic microstructure, photomechanics

## Abstract

The photomechanical response of azobenzene-polymer films to polarized light is sufficiently strong to drive micron-scale surface relief formation. With a multitude of photonics applications, such optically written microstructures have motivated research to identify azopolymers and optical polarization technologies best matched to translate this phenomenon to practical devices. Here we present an overview of this field, with focus on a promising laser-writing platform based on structured polarized light projection from a high-resolution spatial light modulator. This photofabrication approach can print static and dynamic surface microstructures. It is also maskless and single-beam, writing structures with 600 nm feature size and >1 μm amplitude in real-time. The printed structures require no wet-chemical processing and are available for replication immediately after exposure. We also present a new application in color synthesis by printing surface gratings that collinearly diffract red, green, and blue light, demonstrating the potential of this approach in the field of structured color.

## Graphical abstract

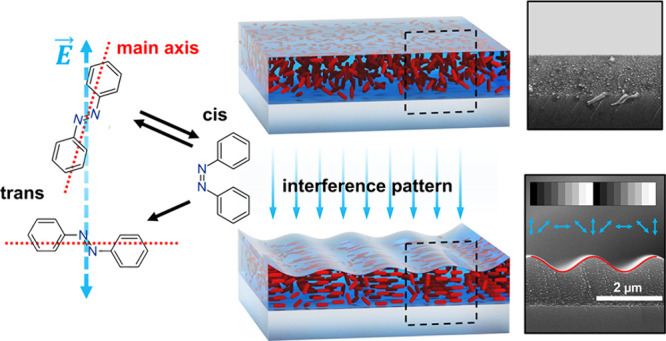

## I Introduction

Since 1995 when it was first observed that azobenzene-polymer films exhibit a photomechanical response strong enough to drive micron-scale surface relief formation, researchers have sought to translate this phenomenon into application.^1–4^ This is particularly evident in photonics, where surface relief microstructures modulate the phase front of an electromagnetic wave, giving them central roles in diffractive optics, information display, and telecommunications technologies, among others. However, until the emergence of high-resolution spatial light modulators (SLM), the ability to project the structured optical polarization fields that would fully exploit the vectorial photomechanical response of azopolymers was quite limited. In parallel, the versatility of supramolecular azopolymers as photonic materials was becoming increasingly clear. Among azopolymers, supramolecular systems in particular offer a modular, tunable, and simple method for creating photomechanical materials. When combined with a spatially addressable polarization field from a spatial light modulator, these systems comprise a new platform for single-beam, real-time optical microstructure fabrication. Here we present an overview of this platform, showing how polarization engineering can elicit an azopolymer photomechanical response that can drive both static and dynamic surface relief structures. We also present new results exploiting the azopolymer polarization response to write surface grating pixels designed to diffractively reconstruct RGB colors, essentially comprising a 2.5D surface relief grating printer.

The outline of this paper is as follows: Section [Sec secII] discusses the fundamental role of photoisomerization along with a brief outline of historical developments including our own contributions. Section [Sec secIII] describes supramolecular azopolymers and their function as model systems for photopatterning, while Section [Sec secIV] provides an overview and comparison of selected supramolecular systems. Section [Sec secV] presents examples from our group of surface micrograting arrays written using two-beam interferometry. Section [Sec secVI] describes our SLM-based system of polarization projection and representative example structures. Section [Sec secVII] outlines an example application of RGB color composition for structured color generation as developed by our group. Concluding thoughts and future directions are presented in Section [Sec secVIII].

## II Photoisomerization and Reorientation: The Polarization Response and Surface Relief Grating Formation

Azobenzene-containing polymers exhibit a strong mechanical response when exposed to certain external radiation fields. These materials can be considered as having two components: an inactive polymer matrix and embedded photoactive azobenzene chromophores.^2^ The radiation field acts only on the photoresponsive azobenzene moieties, which undergo photoisomerization from their stable *trans*- to a metastable *cis*- conformation.^3,5^ Most efficient surface relief grating (SRG) formation was reported for the so-called “push-pull” configuration of the azobenzene groups, where the absorption spectra of the two isomers overlap resulting in a continuous *trans–cis–trans* photoisomerization with a mixed photostationary state under irradiation with a single wavelength.^6^ The molecular properties of both states vary drastically with respect to free volume and dipole moment, and, in fact, one can consider azobenzene chromophores as molecular motors converting optical energy into mechanical work on a molecular time and length scale. The attachment of azobenzenes to a polymer can be achieved in various ways including covalent bonding, as well as noncovalent ionic and supramolecular interactions (e.g., hydrogen and halogen bonding).^7,8^

When a thin azo-polymer film (typically several hundreds of nanometers) is exposed to a spatially varying radiation field, gradients in chemical potential and/or material properties are generated due to spatially varying photoisomerization, which are then transferred to local alignment of the azobenzene side chains relative to the electric field vector. Since the azo-moieties are tied to the polymer matrix, local stresses are generated, leading to macroscopic deformation and the formation of surface relief gratings (Figure 1).^9,10^ This process may run at room temperature (well below the glass transition temperature *T*_g_) and with comparably low irradiation intensities (*I* = 100 mW/cm^2^). The inscription time needed to unlock SRG formation varies between seconds and hours depending on the irradiation parameters and the chemical structure of the polymer. This is very promising for applications^1^ in nano- and microsystem technology such as photolithography,^11^ optical polarizer^12,13^ and waveguide coupler fabrication^14^ or to create lasers, where the emission wavelength is tunable by changing the grating period,^15–17^ as well as mutiplexed grating fabrication for emerging applications in displays.^18^ Besides the optical applications there are a multitude of others, such as guiding templates for living cells,^19^ surface structures with light-controlled wettability,^20,21^ and repositioning of adsorbed nano-objects, just to name a few.^22^

**Figure 1:**
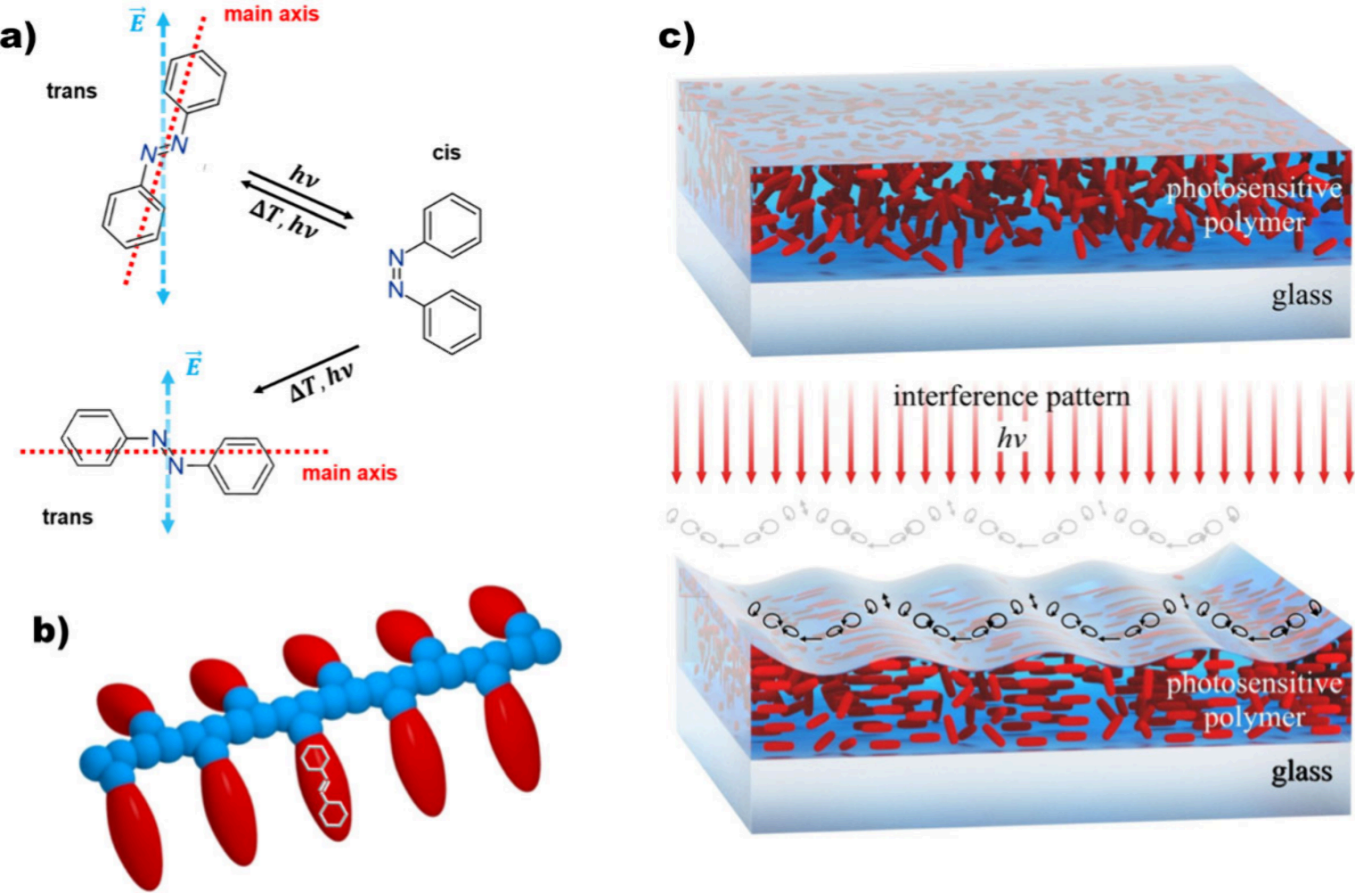
(a) Chemical structure of azobenzene isomers, and scheme of the azobenzene alignment relative to the electric field vector during multiple photoisomerization. (b) Scheme of azobenzene-containing polymer. (c) Scheme of the SRG formation: before irradiation, azobenzene chromophores (in red) are randomly distributed within a polymer matrix (in blue), during irradiation with interference pattern, the local ordering of the azobenzenes results in polymer topography modulation.

Initially, the SRG formation was discovered independently by two research groups in 1995.^23,24^ Even earlier, it was already well-known that linearly polarized light could be used to rotate azobenzene molecules via cyclic isomerization between its *trans* and *cis* conformations. Continuous illumination of the azobenzene molecule at an appropriate wavelength (i.e., typically 400–550 nm) would eventually orient it perpendicular to the linear polarization, at which point isomerization would cease, trapping the azobenzene in a quasi-steady state alignment (see Figure 1a). A distribution of azobenzene chromophores dispersed in a polymer host could therefore be optically transformed from isotropic to anisotropic, with a chromophore order parameter that scales approximately with the intensity. This optical anisotropy (i.e., birefringence) could likewise be spatially modulated by illuminating the film with light of varying intensity and/or polarization generated by, for instance, two interfering laser beams. The resulting birefringence gratings diffract light with a remarkable efficiency, which sparked intense research in holographic and diffractive optics applications.^25–27^ It was eventually established that the local alignment of the azobenzene groups within the polymer film and corresponding SRG formation both contribute significantly to the diffraction efficiency originally attributed entirely to the photoinduced birefringence.^28,1,29,30^ Figure 1 illustrates the main features of photoinduced birefringence and light-induced mass transport.

Experimentally, the conventional method for SRG inscription is by two-beam lithography, where two laser beams of different polarization interfere on the film surface to form either intensity interference patterns (IIP) or polarization interference patterns (PIP). Example configurations (Figure 2) are designated SS, PP, or 45/45 for IIP and SP, RL, or +45/–45 for PIP. Since the light is incident perpendicularly, the electric field vector oscillates in the plane parallel to the surface of the film. In the context of SRG formation, S-polarization is defined as pointing along the grating (meaning it aligns with the periodic lines of the grating), while P-polarization is orthogonal to it. The inscribed topography faithfully mirrors the sinusoidal modulation of intensity and polarization. The resulting deformations can be substantial, with local peak-to-valley variations approaching the total film thickness. For example, a 900 nm grating can be inscribed on a 1 μm polymer film. However, the complete removal of polymer material between the topographical peaks remains unattainable.^31^ The period of the inscribed grating corresponds to the optical period of the interference pattern (IP). The one exception is the PIP corresponding to SP, with the beams S and P polarized, respectively.^32–34^ Here, the grating period can be twice as small as the optical period, thus surpassing the diffraction limit.^35^ Utilizing a combination of two-beam lithography and AFM techniques, it was also possible to correlate the local distribution of the electric field vector within the polymer film with the position of topography minima and maxima (Figure 2).^36^ This resulted in the observation that for most glassy polymers the SRG topography minima appear at intensity maxima for IIP, while for PIP such as RL and +45/–45 the polymer material recedes from the regions of horizontal polarization as defined in Figure 2.^37,38^ The SS optical grating is the least efficient configuration for SRG formation, and can be inscribed only in preoriented films.^39^ In terms of SRG cross-section, the sinusoidal profile common to all configurations of Figure 2 is not optimal for some applications, and can be modified for example to an approximate sawtooth shape by changing the angle between the two interfering beams and/or grating superposition via multiple exposures.^40,41^

**Figure 2:**
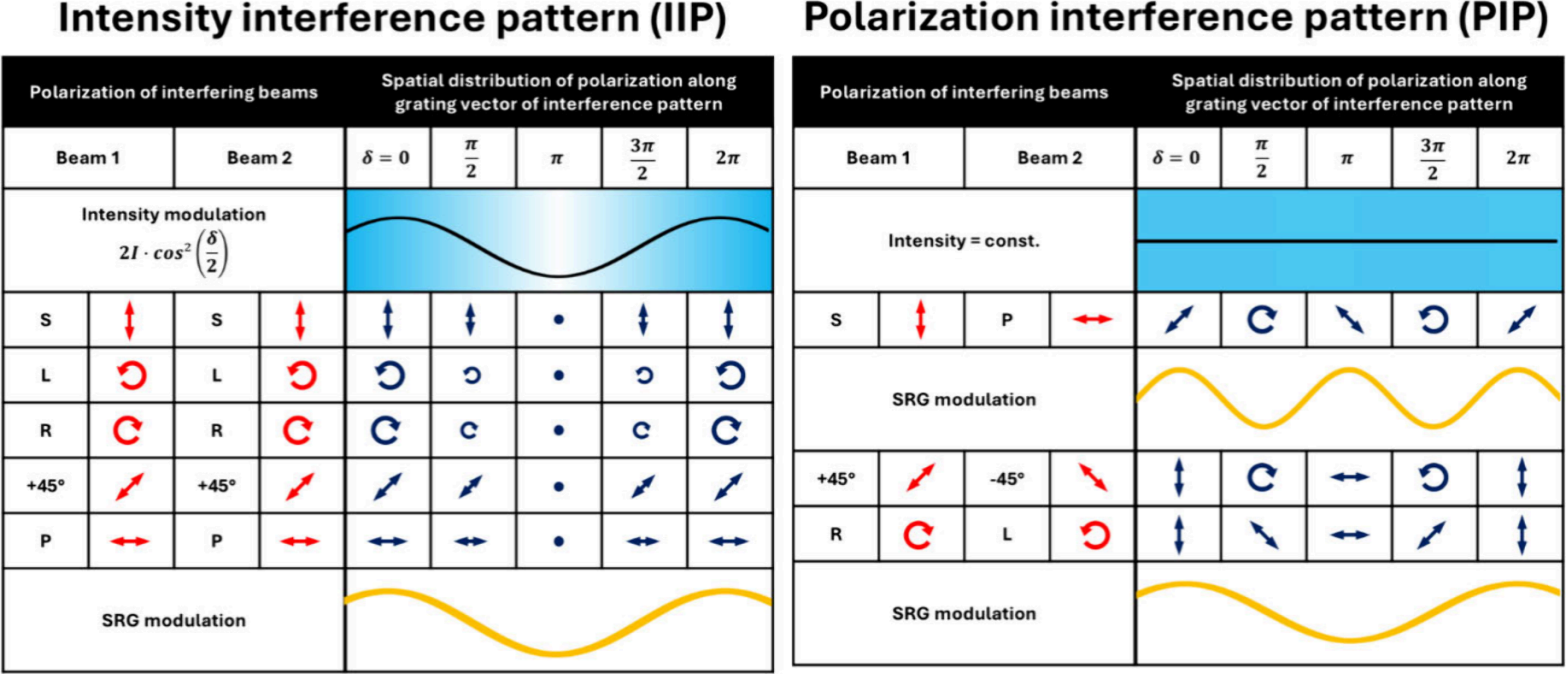
Summary of spatial distribution of the electric field vector and intensity generated during interference of two beams of different polarization. The variation of the topography along an optical period is provided as reported in^31^.

In addition, inscribing structures such as gratings can be achieved not only by far-field irradiation, but also with other types of optical stimuli such as near-field patterns generated by surface plasmon polaritons,^42–47^ with a point-like local illumination through a scanning near-field optical microscope,^48^ or with a single tightly focused laser beam.^49–51^ Utilizing two-beam lithography one can also produce complex patterns by subsequent irradiation with IP rotated at a certain angle with respect to the primary grating.^28,52–54^ Another possibility is to apply four-beam interference patterns also in combination with illumination at multiple wavelengths.^55,56^

As a general feature, the inscribed gratings are stable over years at ambient conditions but can easily be erased or rewritten by, e.g. heating the polymer film above its glass transition temperature, utilizing homogeneous irradiation with circular polarized light, or by changing the phase of the irradiation pattern.^57–60^ Despite all of these possibilities, multibeam lithography is optomechanically limited since the writing wavelength and interbeam angle(s) fix the period and orientation of the resulting structures. Likewise, the projection of complex polarization distributions such as aperiodic, discontinuous, or period-chirped are either impossible or impractical. This has driven the work of our group to explore a different lithographic approach based on the projection of structured polarized light using a spatial light modulator which can in principle generate arbitrary optical patterns, limited only by the device resolution. In Section [Sec secVI] we describe this new approach and show several examples.

Since considerable experimental and theoretical work (summarized in a number of reviews, most recently in refs 61,62,30 ) has already been devoted to the process of SRG formation, it would be natural to assume the process is fully understood. This is however not the case and only recently with the introduction of the orientational approach by Saphiannikova et al., has actual progress been made with respect to relating the orientation anisotropy of the azobenzene side groups to the light-induced mechanical stresses within the polymer films by means of a physical model. This is described in a comprehensive review by Saphiannikova et al.^63^ who addressed the central puzzle surrounding the mechanism of polymer mass transport over hundreds of nanometers within an essentially glassy polymer film (far below *T*_g_) that does not experience significant photoinduced softening.^64^ Informed by this newer understanding, the process of opto-mechanical deformation can be viewed as follows: under irradiation inducing cyclic *trans–cis–trans* isomerization, the azobenzene molecules rotate and reorient perpendicularly to the electric field vector.^65^ This orientational redistribution of the azobenzene moieties subsequently causes a reorientation of the polymer backbones to which they are attached. The macroscopic deformation of the sample is therefore a consequence of the concerted dynamics of backbone segments (Figure 1).^66^ It is quite remarkable that according to this purely mechanical picture of a local, molecular-scale interaction between polymer segment and azobenzene side groups, augmented with a kinetic equation for the relaxation of azobenzene reorientation, it is possible to predict the time-dependent deformation of polymer films^67,64,68^ and the order of magnitude of locally appearing stresses within the films, which was found to be well above the local yield stresses.^69^ Experimentally, the local stress has been measured to be as large as 1 GPa.^70^

## III Supramolecular Azopolymers

Owing to the versatile chemistry of both azobenzene chromophores and polymers and their rich couplings, a broad variety of photoactive functional materials has emerged and is continuously being developed in many fields including chemistry, physics, medicine and engineering.^56^ Depending on the desired functionality one is given many possibilities to design material groups targeted at desired applications. For example, biosystems may require a combination of biocompatibility and excitation wavelengths shifted to the red.^71–73^ For producing optical elements and maintaining thermally stable structures one should utilize polymers of a relatively high glass transition temperature, or even those which have no clear indication of the glass transition temperature.^59,7,74,75^ Extending this to adaptive structures either in optics or surface science, where a change in topography should proceed swiftly and repetitively over many cycles, one would prefer to operate with liquid crystalline polymers.^76–80^

Despite these many reports, and with the newer photomechanical understanding described previously, there remains however no clear understanding of how *chemical* structure affects functional properties of these materials. Apart from the local, single-molecule photoisomerization properties of the chromophores and the number of the azobenzenes moieties incorporated into a polymer backbone,^81–83^ the latent and possibly cooperative response of the underlying polymer matrix must be taken into account to comprehensively understand the phenomenon of opto-mechanically induced structural changes. It is already known, for instance, that how the azobenzene moiety is attached to the polymer backbone with regards to the relative orientation may lead to distinct functional properties.^84^

One of the more pressing questions, however, is of a practical nature: how can we vary azopolymer architectures sufficiently quickly, e.g. without tedious chemical intervention, in order to provide rapid prototypes for intended applications? For this purpose, supramolecular systems are perhaps the ideal material platform. These exploit weak, noncovalent interactions (e.g., hydrogen and halogen bonds, electrostatic interactions, etc.) that, in contrast to covalently linked azopolymers, can be readily synthesized in a single step without elaborate and time-consuming reactions.^7^

## IV Supramolecular Azopolymers for Surface Photopatterning

Several supramolecular azopolymers have been reported in the literature incorporating various azobenzene molecules, different polymer backbones, and different supramolecular interactions. A detailed overview of these systems can be found in a recent review article.^7^ Systematically varying the chemical structure of the azobenzene molecules can provide insight into the observed photomechanical behavior, shedding some light on the structure–property relationship of the supramolecular azopolymers.

We investigated a series of related azobenzene molecules equipped with H-bond donor groups (−OH or -NH_2_) combined with the polymer of choice poly­(4-vinylpyridine) (P4VP) (Figure 3a-g). The hydrogen bond was employed because it is strong enough to couple the azobenzene chromophores to the polymer chain yet flexible enough to support the light-induced orientation of the chromophore perpendicular to the light field vector. This permits some alignment of the polymer parallel to the field in accordance with the photomechanical stress model. Furthermore, the related azobenzene molecules facilitated modulation of the electronic properties and the relative strength of the intermolecular interactions.

**Figure 3:**
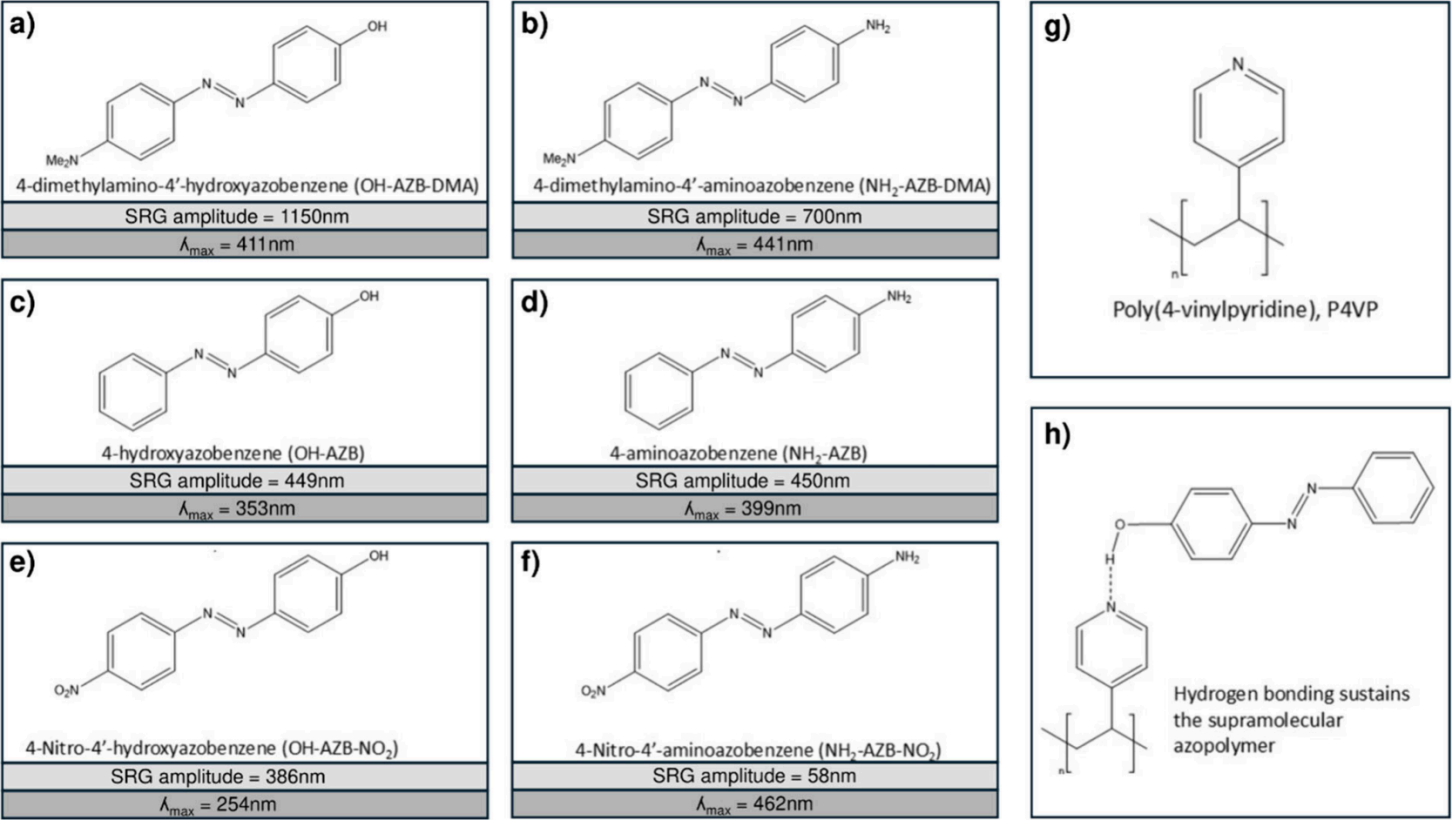
(a-f) Azobenzene building blocks and (g) polymer backbone investigated by the authors. Each azobenzene (a-f) combined with polymer (g) exhibits a unique absorbance λ_max_ and photomechanical response as characterized by the SRG amplitude written using 488 nm light. (h) Example azopolymer of azobenzene (a) and polymer (g) (designated as OH-AZB-DMA-P4VP) used for all imaging and SRG data presented in following sections.

Synthesis of the supramolecular azopolymers was readily achieved by combining DMF solutions of the polymer and the desired azobenzene and stirring the mixture at room temperature for 8 h. Infrared spectroscopy was used to confirm the formation of the supramolecular azobenzene polymer. A shift in the OH-stretch of the azobenzene from over 3000 cm^–1^ to around 2900 cm^–1^, coupled with a shift in the pyridine N=C-stretch from ca. 990 cm^–1^ to over 1000 cm^–1^ is indicative of H-bond formation. Thin films were fabricated by spin-casting the filtered solution on a clean quartz substrate and curing for 24 h at 70 °C. Each azopolymer was then characterized by UV–vis spectroscopy and tested for photomechanical response by exposure to the RL PIP of Figure 2 using 488 nm light. The interbeam angle for the two-beam interference was kept constant, resulting in a 1.5 μm period SRG for each system. The λ_max_ absorbance and steady-state SRG amplitude as measured by atomic force microscopy (AFM) of each system is shown in Figure 3. All systems showed a photomechanical response resulting in SRG growth, with SRG amplitudes from 58 nm up to 1150 nm, highlighting the power of the supramolecular approach and the modularity of the design. By judiciously selecting the molecular components, one can tailor a specific SRG response for a desired application with minimal synthetic effort.

All SRG data presented in the following sections was taken using the OH-AZB-DMA-P4VP system of Figure 3h with a 1:2 complexation, which was originally studied by Vapaavouri et al.^7^

## V Two-Beam Interference: Micrograting Array Fabrication

A common experimental technique to write surface structures on azopolymer films is interference lithography using amplitude-division of the writing beam. Here, two laser beams with a small angle between them overlap on the film surface, as shown in Figure 4a. Typically a single longitudinal mode laser with a Gaussian spatial profile is divided with a beam splitter, and the polarization state of each beam determined with λ/2 and/or λ/4 waveplates to create the desired interference or polarization grating at the film surface as per Figure 2. Another interferometric approach is wavefront-division. This so-called Lloyd’s mirror arrangement consists of a mirror mounted at right angles to the film surface, illuminated with a single writing beam. It is not our intent to review the vast catalog of experimental results obtained with these configurations, but rather to present general observations using the supramolecular class of azopolymers in Figure 3, specifically Figure 3h.

**Figure 4:**
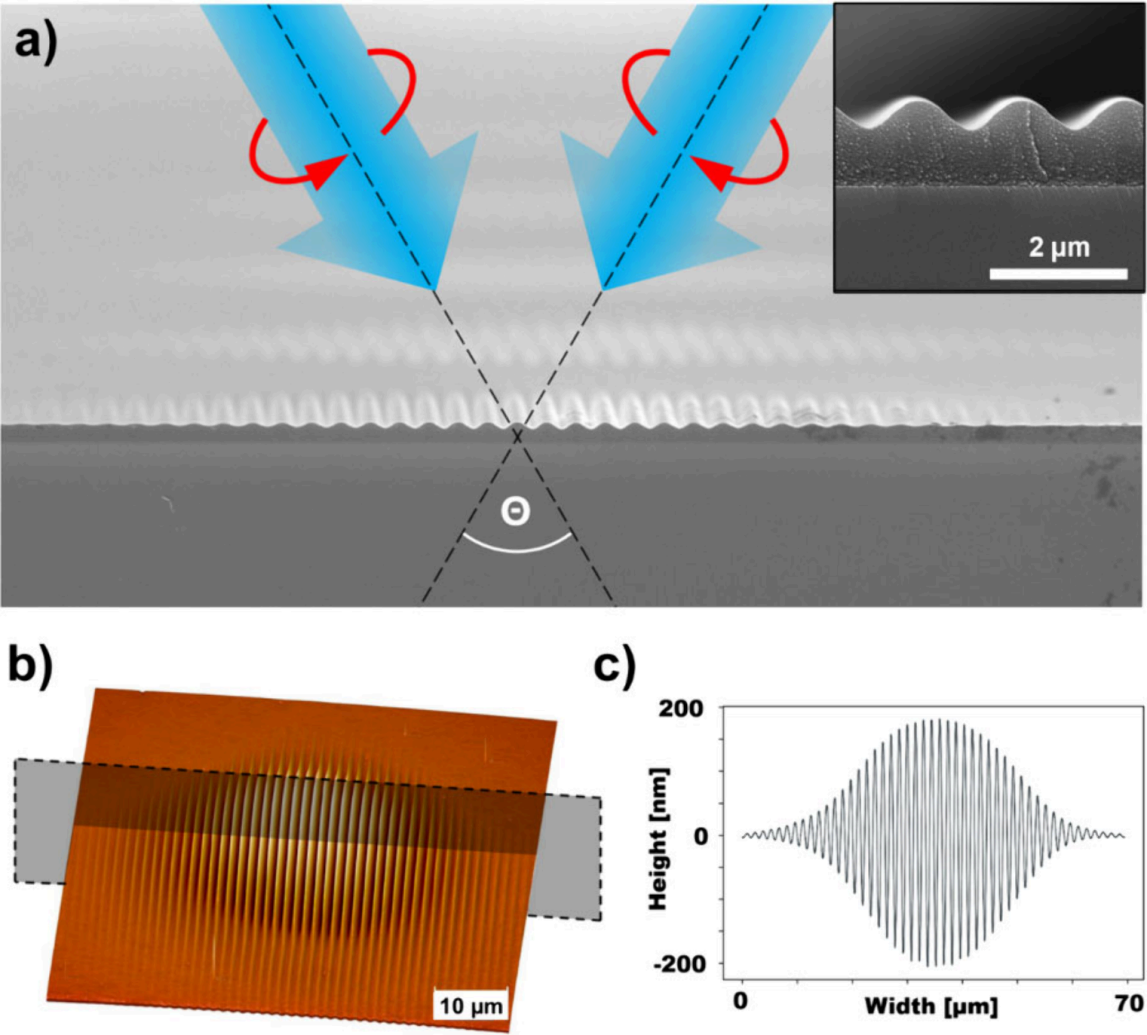
(a) Visualization of the two counterclockwise polarized writing beams, intersecting in the plane of the azopolymer thin film and SEM views of the resulting cross section of the printed SRG. Azopolymer film used is shown in Figure 3h. (b) AFM micrograph of typical SRG structure. (c) Corresponding cross-sectional profile of the AFM micrograph.

The results presented here used two-beam interference (i.e., amplitude division). The laser source is a 488 nm diode-pumped solid state laser. The beam is split into two writing beams, each with a waveplate that delivers the beams onto the azobenzene-polymer film surface with left circular and right circular polarization, respectively. For small interbeam angles, the interference of two counter-rotating circularly polarized beams of wavelength λ and wavevectors *k*_1_ and *k*_2_ generates a linear polarization that rotates across the film surface with periodicity Λ= λ/2 sin (θ/2) and wavevector K_g_. This corresponds to the RL PIP configuration of Figure 2. The resulting surface relief pattern has the same periodicity and will have an amplitude envelope matching the Gaussian intensity profile of the combined writing beams.

A typical experiment using a 1.0 μm thick film involves exposing the film with an intensity of order 100 W/cm^2^ until the sinusoidal surface relief structure reaches a steady-state amplitude, a duration of approximately 10 s. Representative results are shown in Figure 4b,c where the interbeam angle was 18.8 deg, resulting in sinusoidal SRG with period Λ = 1.5 μm. The surface relief grating (SRG) covers a circular area with diameter of 50 μm, which is approximately the 1/e^2^ diameter of the combined writing beams at the film surface. Detailed analysis of SRG amplitude vs exposure time reveals an initial linear growth at 100 nm/s, followed by an approximately exponential approach to steady state, reaching a final amplitude of 400 nm at 10 s. Lower writing intensity results in initial amplitude growth of order 50 nm/s. The actual steady-state amplitude is determined by a combination of photomechanical equilibrium, photodegradation, and film-thickness effects. While focused on the class of supramolecular azopolymers in Figure 3, these results are generally similar in terms of overall behavior of a large class of azopolymers. That is, they respond to light in the 400–532 nm range with surface relief growth on a seconds-minutes time scale, and can reach surface modulations up to 1 μm, and in some cases approaching 2 μm.

While surface relief gratings written with this process function as diffraction gratings, their Gaussian envelope profile and small area limit their applicability. An alternative is to mechanically translate the film between exposures, thus filling a macroscopic area with individual gratings. Such two-dimensional micrograting arrays can have the amplitude and subsequent diffraction efficiency of each element controlled by the laser exposure time. These arrays have applications as outcouplers for backlight and display systems, as optical diffusers, and in optical security as anticounterfeit devices.^85–89^

One demonstration illustrating this approach is the transformation of a grayscale digital image to a corresponding dot-matrix of surface relief gratings, where the value of each pixel is mapped to the amplitude of a corresponding surface relief grating on the film via the exposure time. Since diffraction efficiency scales with grating amplitude, the perceived brightness of a pixel when viewed at the m = +1 diffraction angle will be representative of the pixel value of the original image. As an example, consider the image of Albert Einstein in Figure 5a. By multiplying the value of each pixel by 0.01 s, we obtain a simple mapping of the pixel value to grating amplitude, appropriate for our supramolecular polymer film, beam size, and optical writing intensity.

**Figure 5:**
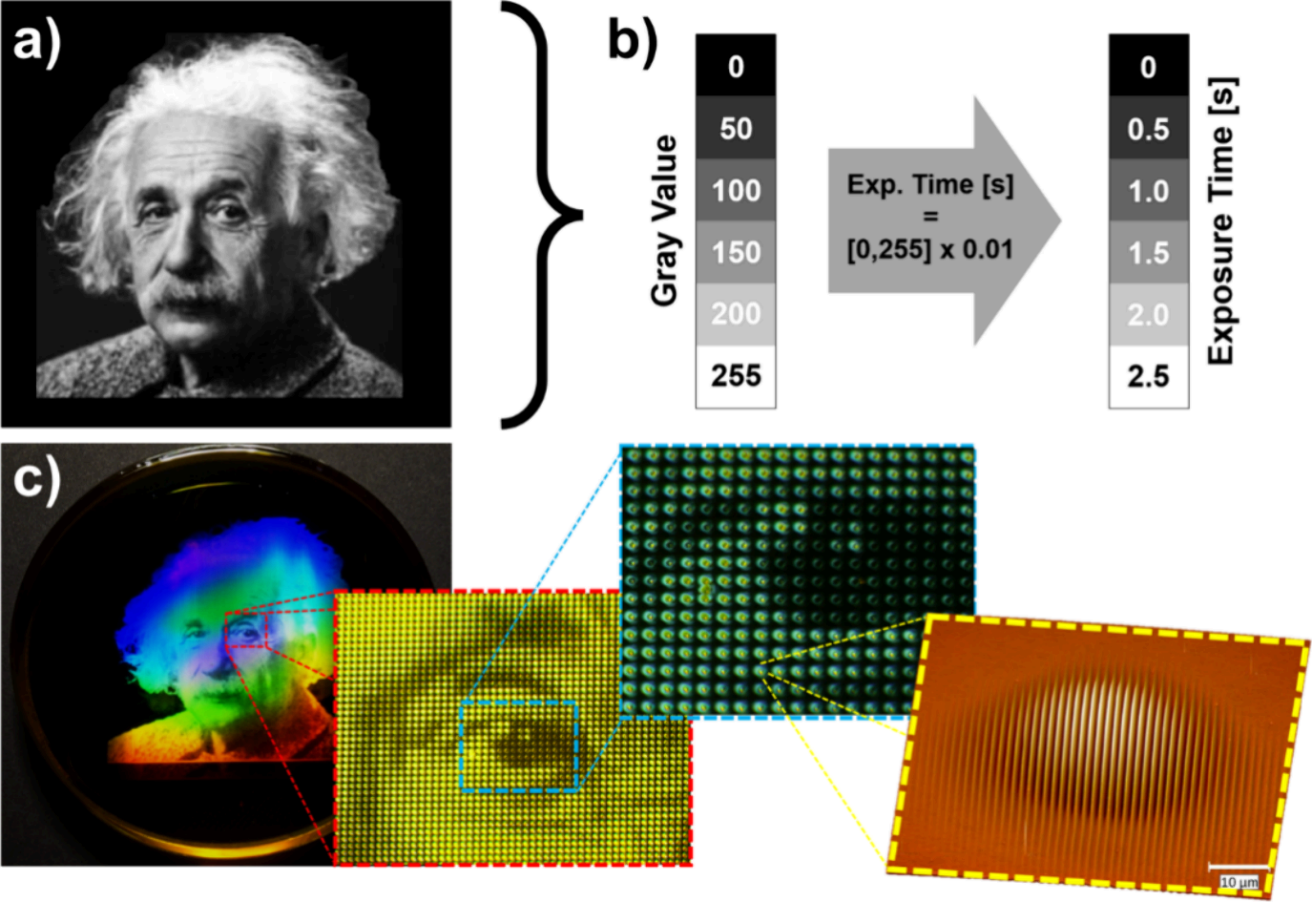
(a) Grayscale image of Albert Einstein. (Turner, O. J., photographer. (ca. 1947) Albert Einstein, 1955, Courtesy of the Library of Congress Prints and Photographs Division, LC-USZ62–60242) (b) Grayscale mapping scheme, translating 256 gray values to exposure time from 0 to 2.5 s. (c) From left to right: photo of dot-matrix hologram under white light; low magnification microscopic view; high magnification microscopic view; AFM micrograph of single SRG dot. Reproduced from ref 90. Copyright 2021. Society of Photo-Optical Instrumentation Engineers (SPIE).

From the original 235 × 235 JPEG image, a corresponding 235 × 235 surface relief grating array was printed and viewed at the m = +1 diffraction angle, as shown in Figure 5c. The image is viewed in reflection using white light illumination. The exposed area is approximately 15 × 15 mm^2^, giving a resolution of 390 dots per inch (dpi). Each surface relief grating (i.e., dot) has period 1.5 μm with its wavevector oriented vertically, resulting in the brightly colored image when viewed as shown. Rotating the film 90 deg in the plane of the page causes the image to disappear. Likewise, by keeping the film fixed in the plane of the page and sweeping the viewing angle across the grating planes reveals again a multicolored image that rapidly changes in color as a function of that viewing angle.

These micrograting arrays, also referred to as dot-matrix holograms, can be viewed immediately after exposure and are stable under ambient light, temperature and humidity, with no observable changes in brightness 3 years after fabrication. As a surface microstructure, the dot-matrix is easily replicated using well-known nanoimprint techniques such as PDMS molding.

## VI Microstructure Fabrication Using SLM-Based Digital Polarization Optics

From 1995 through 2021, nearly all experimental studies of surface relief grating formation in azopolymer systems were carried out with some version of the two-beam interference setup described in Section [Sec secV]. Two limitations of this approach are that the SRG has a Gaussian amplitude profile and that the area of the SRG written is the area of the intersecting laser beams on the film surface. Another is the need for long coherence length lasers and a mechanically stable environment. Perhaps most limiting is that a given writing arrangement produces an SRG of a single, fixed periodicity and orientation, which can only be changed through optical realignment and/or a different wavelength laser. More fundamentally, two-beam interferometry cannot generate the spatially aperiodic polarization necessary to not only probe the azo-polymer interaction but also to write the diversity of surface microstructures required for applications.

To overcome this, McGee et al. developed in 2022 a different approach based on the projection of spatially structured polarized light.^91^ In short, a linearly polarized laser beam passing through a birefringent liquid crystal cell placed between crossed quarter waveplates will rotate the polarization plane depending on the liquid crystal retardance. This can be implemented on a pixel-by-pixel basis using a spatial light modulator, thereby enabling the projection of linear polarization fields with programmable directionality. Combining this with the extraordinary polarization sensitivity of supramolecular azopolymers has resulted in a new optical microstructure fabrication platform with particular versatility in writing complex, aperiodic structures in a single exposure, dynamic “conveyor-belt” surface waves,^92^ and surface-relief color pixels for programmable RGB structured color applications.

Our approach was motivated by researchers using the SLM to write surface-relief structures on azopolymer films through activating an intensity-based photoresponse.^93–95^ Unique to our approach has been configuring the SLM as a pure-polarization modulator. Here, the optical intensity is spatially uniform and it is the spatially varying polarization that drives the material photomechanical response. This concept of digital polarization optics was introduced in ref 96 in experiments writing local optical axes in an azobenzene-based film used as a liquid crystal photoalignment layer.

The SLM used here is a liquid crystal on silicon reflective device (Meadowlark Optics) with 1920 × 1152 pixels. It is operated in a so-called polarization rotator configuration between two crossed quarter waveplates (see Figure 6). In this way, an incoming linear polarization can be transformed into a complex polarization pattern. Each pixel rotates the incoming vertical polarization by approximately 1.4 deg per gray level. With 255 gray values (8 bits) available, a full 360 deg rotation of the polarization can be achieved.

**Figure 6:**
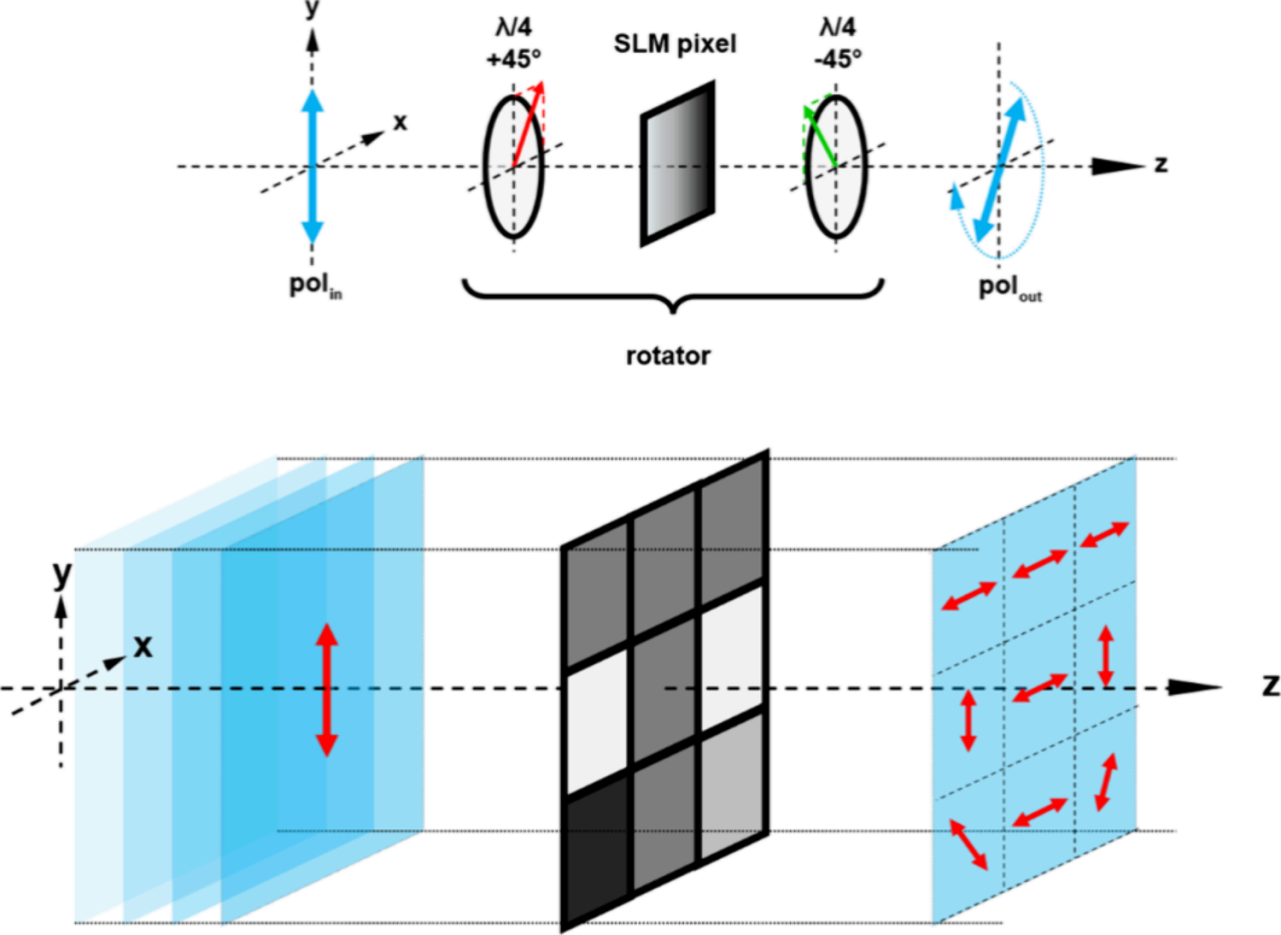
Top: working principle of the SLM polarization rotator configuration; optical retarder (SLM pixel) between crossed quarter waveplates with incoming linear polarization and outgoing, rotated polarization depending on the addressed gray value. Bottom: scheme of a pixel array in rotator configuration with incoming vertical, linear polarization and outgoing complex polarization pattern. Reproduced from ref 92. Copyright 2023 Wiley-VCH.

A schematic of the SLM setup is shown Figure 7. The light source is the same laser as in the two-beam setup (Coherent Sapphire 488 nm). The beam is expanded with a telescope to illuminate the entire area of the SLM (∼2 cm^2^), and the quarter waveplates (λ/4) before and after the SLM comprise the polarization rotator configuration. The image of the SLM is projected onto the film using a 40X microscope objective, which in our setup produces an image size 130 μm by 80 μm. A dichroic mirror in the beam path and an off-axis camera are installed for targeting and in situ observation of the SRG writing process. The film is mounted on motorized xy stages for raster scanning of larger areas.

**Figure 7:**
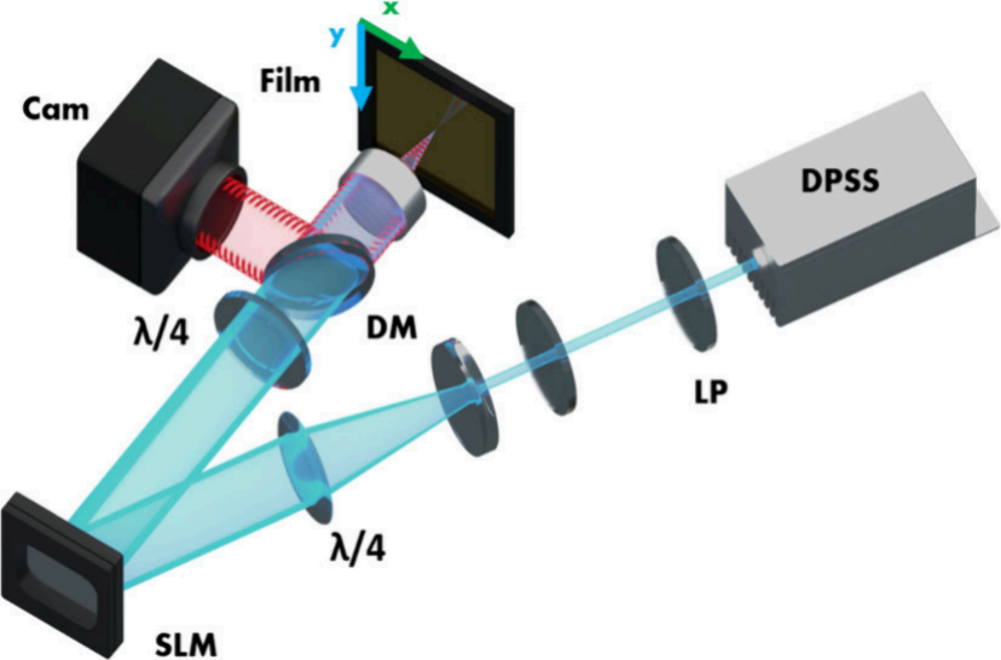
Scheme of SLM-setup with a diode pumped solid state laser (DPSS 488 nm), rotator configuration (λ/4–SLM−λ/4), in situ observation (dichroic mirror + camera), microscope objective and azopolymer film on xy-stage.

Figure 8 shows AFM images of sample microstructures printed with the SLM system using a single 5 s exposure. The top two images (8a,b) are a SRG with sinusoidal profile with a 2.0 μm period and approximately 400 nm peak-to-peak surface modulation. This was created by addressing the SLM with a periodic grayscale pattern (shown in the overlay) that at the film surface produces the same RL PIP polarization grating as shown in Figure 2. While the AFM images in 8a,b are over a 20 × 20 μm^2^ area, they are representative of the entire 130 μm x 80 μm exposure area on the film. This amplitude uniformity over the entire exposure field is clearly one immediate improvement over the two-beam interferometric approach. The bottom images (8c,d) demonstrate a more powerful advantage: namely, that arbitrary surface relief structures can be written in a single exposure simply by addressing the SLM with the appropriate grayscale pattern, which is subsequently converted to an optical polarization field at the film surface.

**Figure 8:**
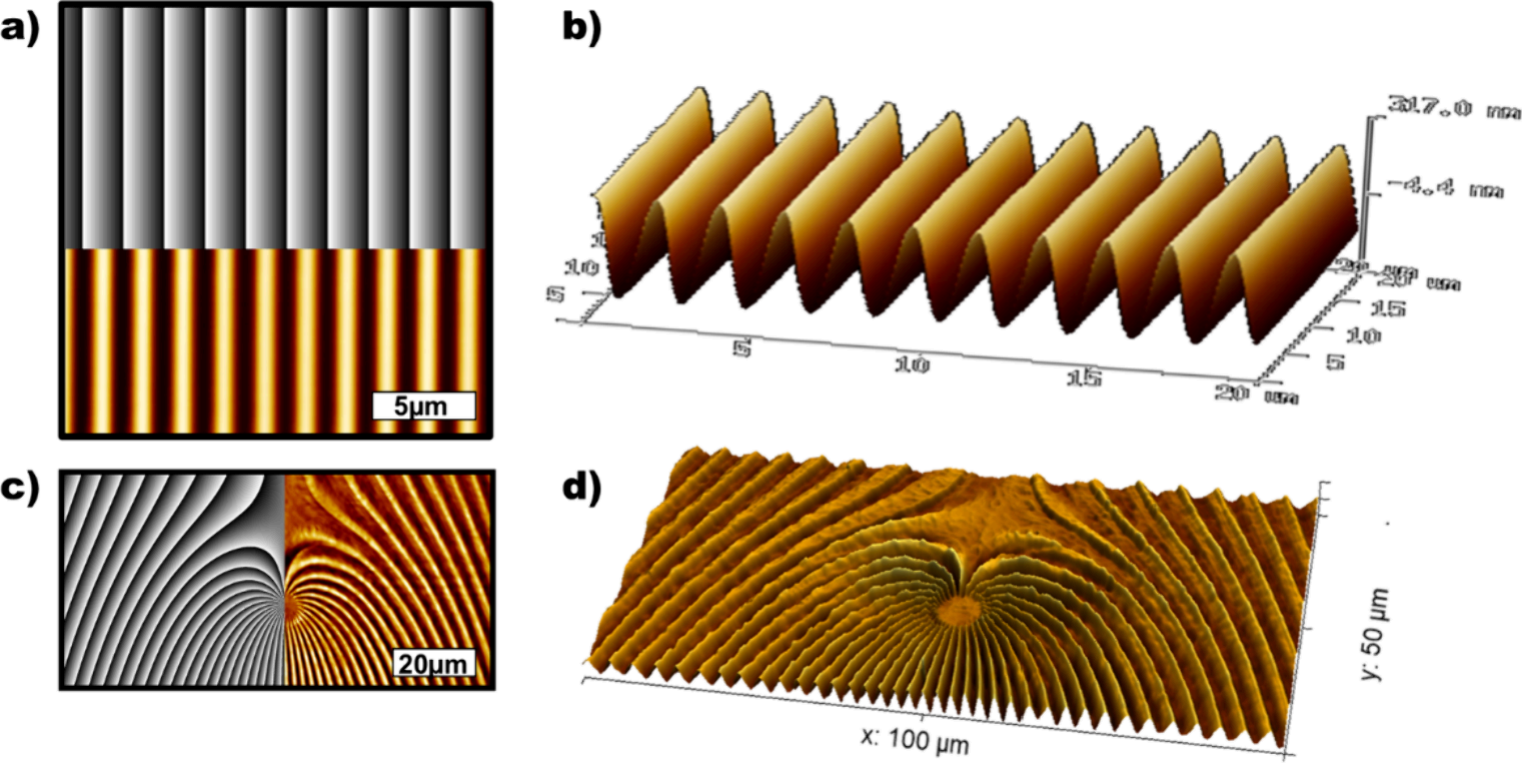
Surface relief structures created with SLM setup in Figure 7. (a) 20 × 20 μm^2^ 2D AFM micrograph of a linear grating of 2 μm periodicity (scale bar = 5 μm; amplitude 320 nm) with corresponding SLM grayscale pattern overlaid in the top half. (b) 3D AFM map of this SRG. (c) 100 × 50 μm^2^ 2D AFM micrograph of a more complex SRG (scale bar = 20 μm; amplitude 430 nm) with corresponding SLM grayscale pattern overlaid on the left half. (d) 3D AFM map of the complex SRG. Typical optical intensity at film plane is 90 × 10^3^ mW/cm^2^, with exposures of order 5 s.

To demonstrate the versatility of this approach, consider Figure 9 which shows how simple diffractive optical elements (DOE) can be embedded in larger SRG grating arrays to create for example sophisticated optical security elements generally referred to as diffractive optically variable image devices (DOVID). First, consider that the amplitude, period Λ, and orientation of each 120 μm x 80 μm SRG element on the film surface can be independently defined with the appropriate grayscale pattern on the SLM. This enables a far wider palette by which pixel information can be mapped to surface structure than in conventional two-beam interferometry. Figure 9a shows an example film photopatterned with a 62 × 106 array of surface relief gratings, each of area 120 μm x 80 μm. This array contains a DOE constructed of elliptically shaped gratings designed to generate in transmission an image of atomic orbitals while the remaining gratings in the matrix show in reflection an image of a light bulb surrounded with spectral colors. Figure 9b is an optical micrograph of the film surface, showing a representative sample of some of the SRG matrix elements. The fabrication is computer-automated, with the user designating the desired DOE far-field image, the desired reflected image, and the number of SRG grating elements on the film.

**Figure 9:**
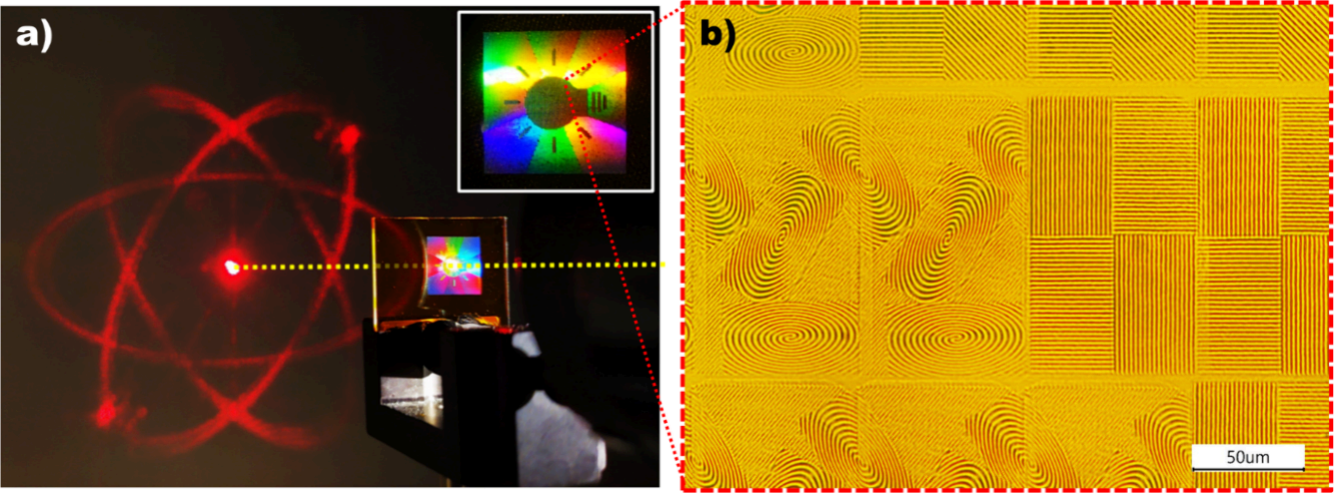
Surface relief micrograting array with DOE structure in the center. (a) Upper right inset: Surface relief array viewed in reflection under white light illumination. Collimated red laser transmitted through the central DOE area (yellow dotted line) generates far-field pattern of atomic orbital. (b) Optical micrograph of a central region of the dot matrix hologram. Reproduced from ref 97. Copyright 2023. Society of Photo-Optical Instrumentation Engineers (SPIE).

### VI.I Aperiodic Surface Microstructures

Another unique capability of the SLM approach is the inscription of aperiodic structures which cannot be accomplished with two-beam interferometric methods. Consider for example Figure 10a which shows a binary grayscale image of Albert Einstein. Because it is a binary grayscale, there are regions where black and gray meet, which when addressed to the SLM, ultimately produce a region on the film where the optical polarization abruptly changes from horizontal to vertical. At such locations we observe the largest surface deformation, whereas in the regions of uniform polarization (i.e., all black or all gray) we see no deformation. This capability is particularly useful in furthering understanding of the complex relationship connecting surface deformation with the underlying gradients in azobenzene and polymer chain orientation.

**Figure 10:**
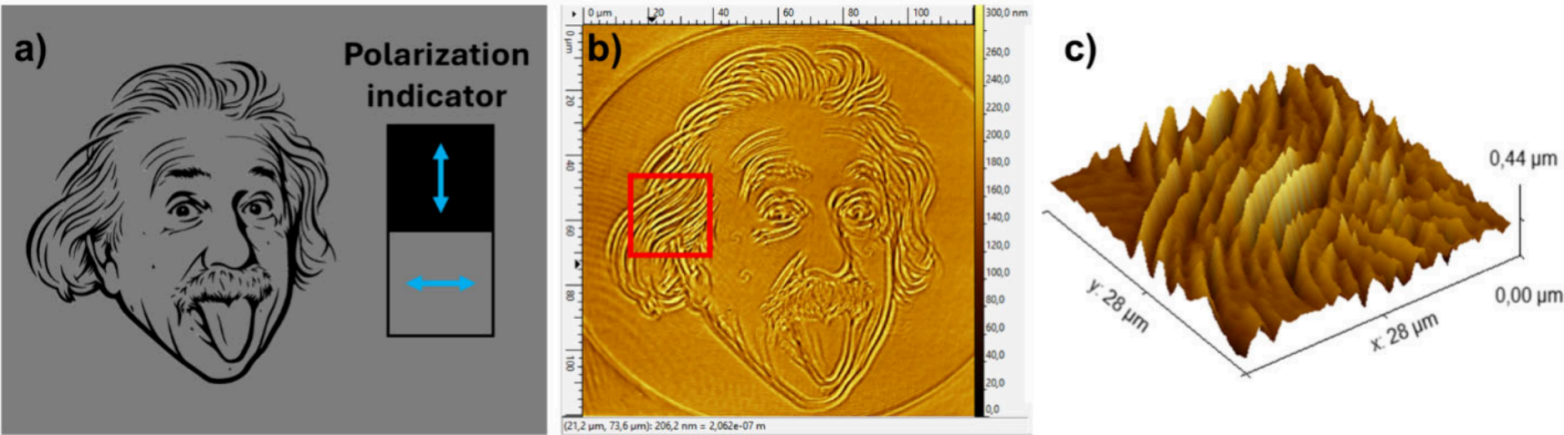
(a) Binary grayscale image of Albert Einstein^98^ addressed to SLM. Black corresponds to vertical polarization and gray corresponds to horizontal polarization. (b) AFM micrograph of resulting surface relief structure on azopolymer film following 20 s exposure. (c) Zoomed in 3D view of indicated area in (b).^98^

### VI.II Dynamic Surface Microstructures

In addition to the facile photofabrication of static microstructures, a fundamentally distinguishing characteristic of azopolymers is that their photomechanical response enables the generation of dynamic microstructures driven by light. This has been demonstrated using two-beam interferometric methods in which the optical field and/or film are translated during exposure,^99^ and more recently by our group using the SLM-based approach. In terms of dynamics, the advantages of this approach over two-beam interferometry are perhaps even more apparent because all that is required is to animate the grayscale pattern addressed to the SLM. That moving grayscale maps to a moving polarization distribution on the film surface, enabling the generation of traveling surface relief gratings, including subregions within the SLM frame, each moving with an independent velocity, direction, and periodicity.

In the initial “write” phase, the SLM is addressed with the grayscale function (shown in blue in Figure 11a) creating a surface relief pattern on the film with peak-to-peak modulation of d_pp_ and period Λ. The pattern is then phase-shifted by +Λ/2 (shown in red), starting an “erase” phase. Following erasure of the surface structure, the SLM pattern is not shifted but continues to expose the film, “rewriting” the erased structure shifted in phase by +Λ/2. The SLM pattern is then shifted −Λ/2 to repeat the process (not shown in the figure). The experimental demonstration of the first write-erase-rewrite phase is shown in Figure 11b, where the top 1/3 of the SLM frame is not animated so as to provide a spatially fixed reference structure. Figure 11c is an AFM scan at t = 2.0 s showing the structure after the initial write phase, and the AFM scan at t = 5.0 s showing the rewritten structure shifted by Λ/2.

**Figure 11:**
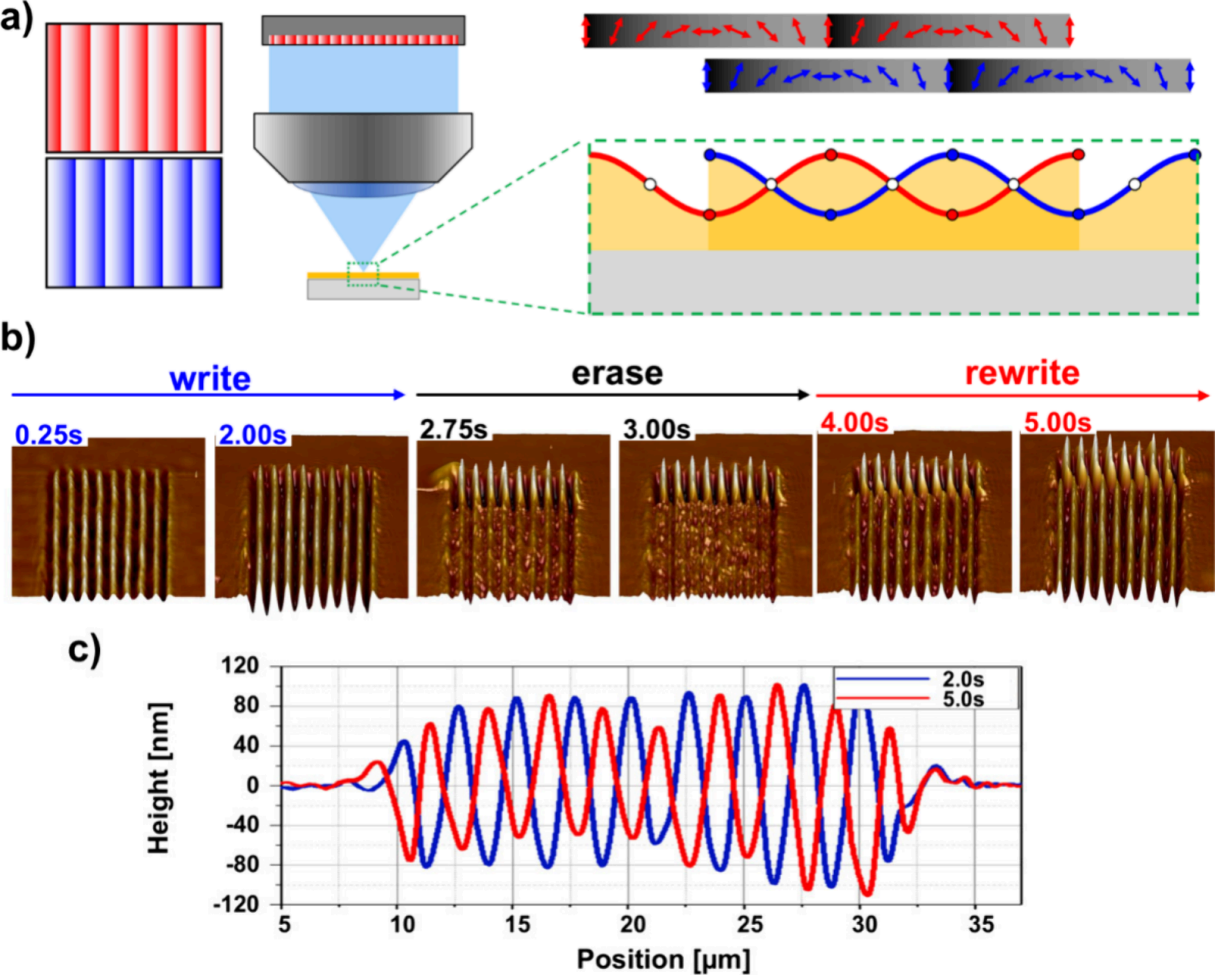
Demonstration of the reversibility of the material deformation by generating a standing wave in the topography of the azopolymer thin film. (a) Schematic of the two polarization patterns addressed on the SLM (left) and corresponding polarization distribution and material deformation on the right. (b) AFM micrographs taken after certain time intervals, showing the two seconds of writing phase, two seconds of erasure and one second of rewriting. (c) AFM profiles after two seconds of writing (blue) and after additional three seconds with shifted polarization pattern, leading to a rewritten SRG shifted half a period (red). Reproduced from ref 92. Copyright 2023 Wiley-VCH.

This process can be repeated, creating a purely optically generated microstructure that behaves effectively as a standing surface wave, with d_pp_ = 170 nm and nodes separated by Λ/2. Similarly, to generate a traveling surface microstructure, the initial pattern of Figure 11a (blue) is simply animated to move continuously to the right or left.

Depending on the application, such write-erase-reconstruct functionality can be essentially quasi-static. This would benefit applications in bioengineering, such as for example the use of synthetic microstructures as templates for directed cell growth.^100–102^ The time scales for in situ surface reconfigurations in this context are particularly well suited to the azopolymer-SLM system presented here.

## VII Structured Color with RGB Surface Relief Gratings

One new application well-suited to leverage the programmable periodicity of this laser-writing process is structured color. This refers to color generation from a geometric interaction between light and matter in contrast to a photochemical interaction such as absorption by dyes. Structured color offers next-generation potential in color-specific technologies such as anticounterfeiting and information display in part because it is a high-brightness and fade-resistant approach.^103–107^

Structured colors are readily generated by diffractive processes. Here, the wavelength separation produced by the surface geometry is engineered to produce collinear propagation of separate colors. For example, consider 3 surface relief gratings each with a period d_r_, d_g_, and d_b_ such that λ_r_/d_r_ = λ_g_/d_g_ = λ_b_/d_b_ and where λ_r_ = 0.633 μm (R), λ_g_ = 0.532 μm (G), and λ_b_ = 0.488 μm (B), all illuminated at normal incidence with broadband light. In this case, the m= +1 mode from the 3 gratings will consist of diffracted colors R, G, and B, copropagating along a common direction θ determined by the ratio of wavelength to period. Recent examples of this approach using two-beam interferometric writing on azopolymers can be found in ref 108 and 18.

Using the setup in Figure 7, writing the 3 RGB gratings on the azopolymer film is accomplished in a single exposure by subdividing the SLM frame into 3 regions of period d_r_, d_g_, and d_b_ and area A_r_, A_g_, and A_b_ respectively. By selecting the three periods to diffract the m = +1 modes collinearly, the respective diffracting areas, and not the grating amplitude, will define the proportional red, green, and blue intensities. In the far field an observer will perceive the superposition color, allowing for the generation of custom diffractive colors simply by varying the relative areas A_r_, A_g_, and A_b_. This method of additive color generation is similar to the digital 8-bit per channel RGB color representation system used in display devices, wherein color is constructed over the (0,0,0) through (255, 255, 255) range. Black (0,0,0) corresponds to the absence of light, and the device-dependent white corresponds to equal components of R, G, and B, each in the fully on (255, 255, 255) or maximum intensity state.

An experimental demonstration of this functionality is shown in Figure 12. Consider the color white which in the RGB system is defined as (R,G,B) = (255, 255, 255). To approximate this color using a diffractive approach, the SLM frame is divided into areas proportional to the R, G, B weights (33.3, 33.3, and 33.3 respectively) as shown in Figure 12b. We then select a viewing angle for the m = 1 mode (e.g., 14 deg from normal) which subsequently defines the period of each region as d_r_ = 2.59 μm, d_g_ = 2.18 μm, or d_b_ = 2.0 μm, respectively. To define brightness, we draw from practical experience wherein laser exposures from 0 to 2 s write gratings of minimal amplitude 10 nm up to a maximum of approximately 300 nm. For a given film composition, this range depends on the laser power and image size of SLM frame projected at the film surface, which here is 240 μm x 145 μm. With these parameters, the film is exposed sequentially from left to right, starting at 2 s. Following this 2 s exposure, the film is translated 240 μm and exposed for 1.98 s. This process is repeated for 90 exposures, ending with 0 s exposure on the right. In the next 6 rows, the process is repeated for red, green, blue, cyan, magenta, and yellow. Figure 12a is a JPEG construction of this color map and Figure 12c shows the exposed film viewed at the designed 14 deg viewing angle when illuminated at normal incidence with white light. The diffractive colors approximate the original JPEG color map. Color shifts are expected since the relatively large area of the exposed film subtends an angular range centered at 14 deg at the camera.

**Figure 12:**
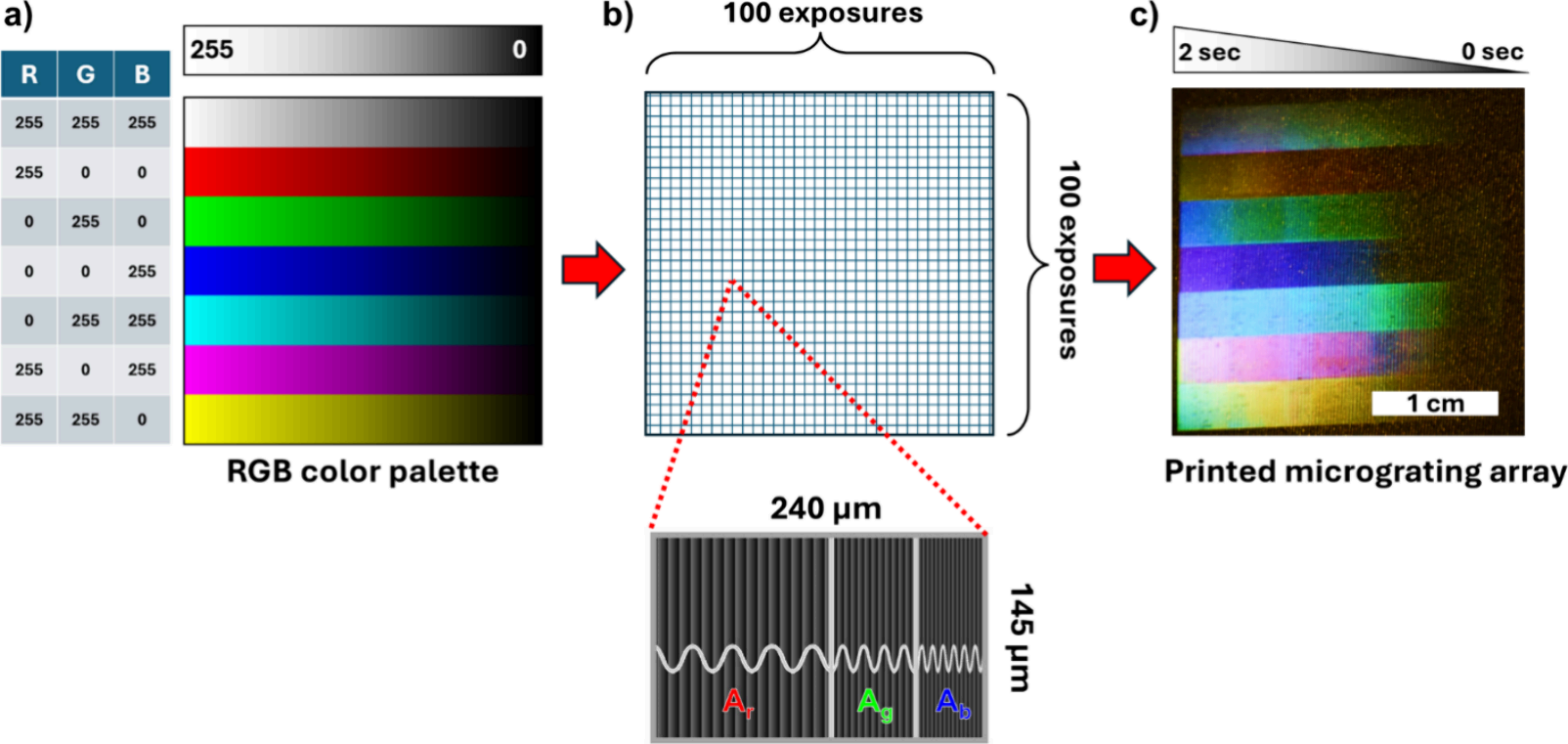
Experimental demonstration of RGB color superposition with diffractive structured color. (a) Digital 8-bit per channel RGB color representation of white, red, green, blue, cyan, magenta, and yellow. (b) Schematic illustration showing subdivision of SLM frame into respective R, G, and B regions such that λ_r_/d_r_ = λ_g_/d_g_ = λ_b_/d_b_ where d is the period of the respective region. Color superposition is achieved through programmable definition of areas A_r_, A_g_, and A_b_. Although 3 distinct regions are shown, this is to illustrate the most general case, and is not intended to refer to the specific pixel indicated. (c) Azopolymer film following exposure, viewed at 14 deg from normal and illuminated at normal incidence with broadband light.

Having recently demonstrated this method of creating structured color with diffractive surface gratings, we present newer results here showing how this can also be used to transform color digital images to an equivalent representation in micrograting array form. In the simplest method, the pixel-by-pixel RGB information in a typical image (e.g., in JPEG form) is extracted and converted to an equivalent subdivision by area of the corresponding SLM frame. In other words, each image pixel is mapped to a corresponding 240 μm x 145 μm pixel which is subdivided into 3 gratings that proportionally diffract the R, G, and B components of the original image pixel. To map the corresponding brightness, the exposure time (which determines the grating amplitude) is scaled to the sum R+G+B for each pixel.

An example of this technique applied to a 60 × 90 image of the Mona Lisa is shown in Figure 13. The image was first transformed pixel-by-pixel into a 60 × 90 array of grayscale patterns containing the appropriate R, G, and B areas, along with a corresponding exposure time. This information was then sequentially addressed to the SLM in Figure 7, with the azopolymer film being translated in an XY raster pattern between exposures. The resulting micrograting array was illuminated with white light at normal incidence and viewed at the designed 14 deg viewing angle, as shown in Figure 13c.

**Figure 13:**
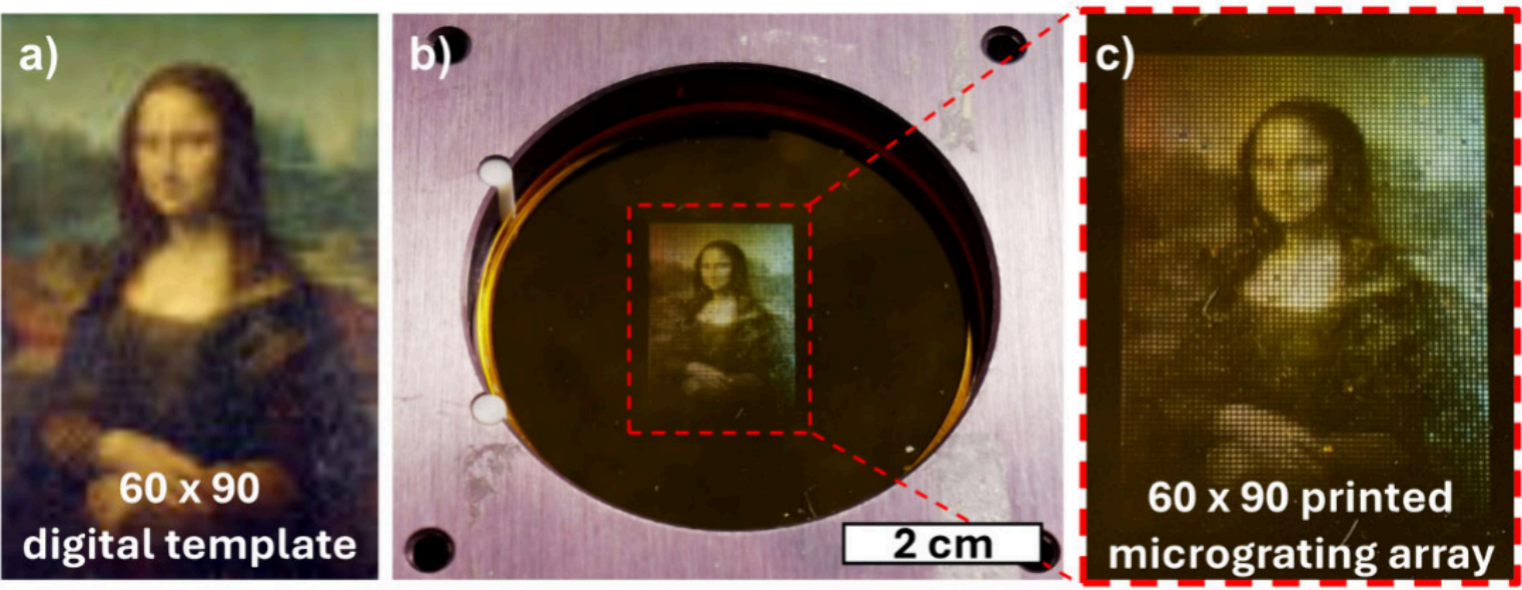
(a) Original Mona Lisa^109^ at 60 × 90 resolution (b) Dot-matrix representation printed as 60 × 90 micrograting array on azopolymer film. Each pixel is 240 μm x 145 μm and is subdivided into RGB regions as per Figure 12. Film is illuminated at normal incidence with broadband light and viewed at 14 deg from normal.

To demonstrate the versatility of this programmable surface-relief grating printer, consider that the resolution can be enhanced by further subdividing the grayscale pattern addressed to the SLM into 6 subpixels, with each subpixel containing the appropriate R, G, and B diffracting areas. Using this approach, a 180 × 270 image of the Mona Lisa (Figure 14a) was transformed into a micrograting array (Figure 14b,c), where the enhancement in resolution over Figure 13 is clearly evident. For all such micrograting array images, a photograph at the m = +1 location (in this case, 14 deg from normal) where color superposition occurs does not fully capture the brightness or angular selectivity associated with structural color. In Supporting Information a video shows the Mona Lisa micrograting array of Figure 14 as it is rotated with the camera in a fixed location, demonstrating clearly the appearance of the color image over only a very narrow angular range.

**Figure 14:**
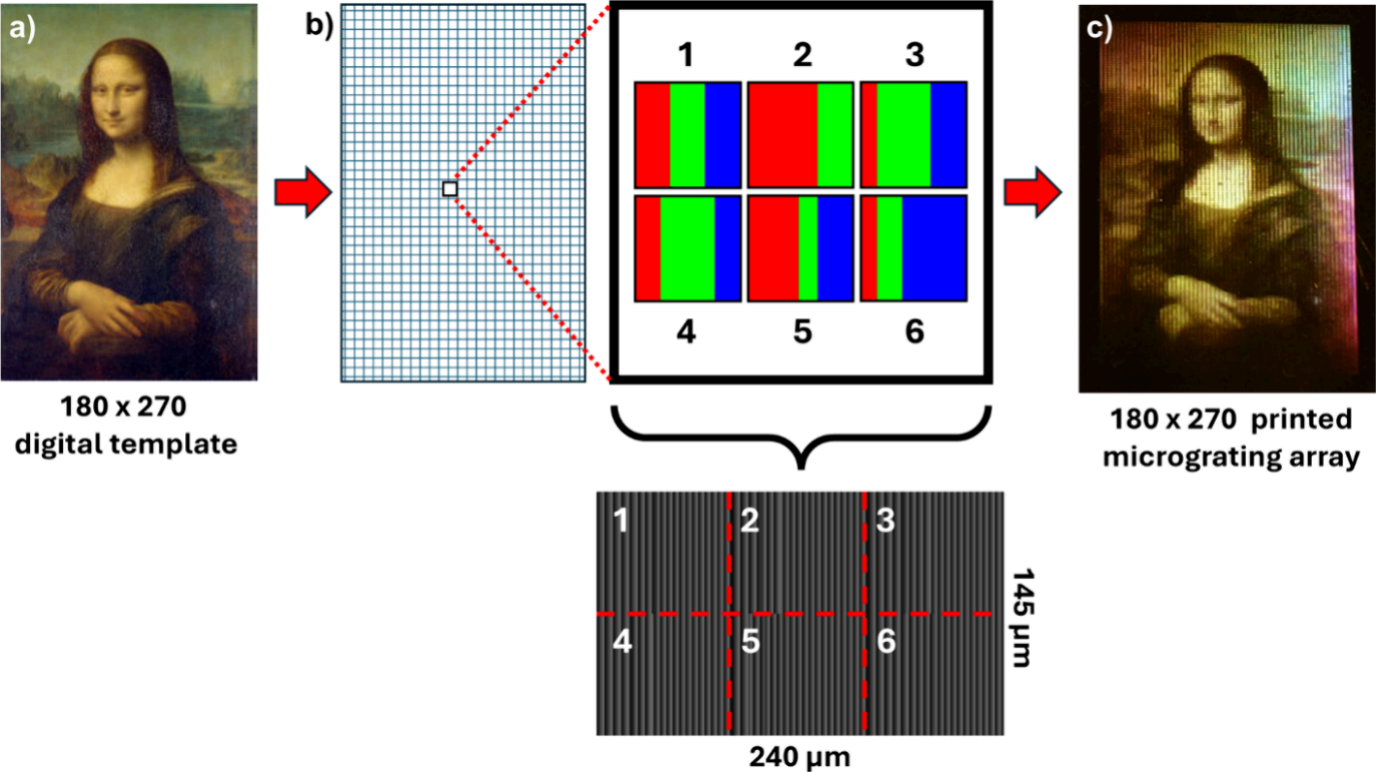
(a) Original Mona Lisa^109^ at 180 × 270 resolution. (b) Schematic illustration demonstrating resolution enhancement via subdivision of SLM frame into 6 subpixels. (c) Exposed azopolymer film viewed at 14 deg when illuminated with normally incident broadband light.

This technique of printing color images as diffractive micrograting arrays extends to multiple applications, including as diffractive optical elements and as templates for bioengineered surfaces. As diffractive components, such arrays are integrated in LED backlight systems, automotive illumination, and optical security devices.^85–89^ In bioengineering, synthetic microscale surface gratings are used to influence cell response, including directed cell growth.^110–112^ In addition, there is no a priori reason why surface gratings must be stitched together via multiple exposures. When transforming a digital image pixel-by-pixel to a similar pixel-by-pixel array of surface relief gratings, this stitching approach is natural. However, the film and/or writing beam can be continuously translated perpendicular to the grating vector, essentially painting a surface relief grating in real time. We have recently demonstrated this approach to structured color generation in ref 113.

## VIII Summary and Outlook

We have presented an overview of the evolving field of surface patterning using photomechanically responsive azopolymer films, with emphasis on supramolecular azopolymers as the print media and digital polarization optics as a maskless and single-beam laser writing technique. This photofabrication platform can print feature sizes of order 600 nm and surface amplitudes up to 1 μm, with printable surface area 250 μm^2^ for a single exposure, increasing up to 300 mm^2^ and beyond for stitched or continuous exposures. For periodic structures such as surface relief gratings, the amplitude, period and orientation are controllable via SLM programming with the appropriate polarization map, with no optomechanical adjustment required. In dynamic mode, surface relief grating (SRG) waves can be generated, at speeds up to 1 μm/s simply by addressing the SLM with the appropriate dynamic grayscale map.

The photomechanical response of the azopolymer enables many of the key advantages of this material as a print media for surface microstructures. Because it is slow compared with the response of photochemical materials (e.g., photoresist), many of the experimental complexities inherent to holographic inscription are mitigated here. High frequency vibrations are essentially ignored, and the writing process can be visualized in real time in ambient lighting, with the surface relief structure available for replication immediately after printing. Somewhat less recognized is that by using a vectorial (i.e., polarization-based) response in the combined azopolymer-SLM writing system, the most significant demand for optomechanical stability rests on the projected spatial polarization pattern, and not on a two-beam interference pattern based on interferometric beam division. This stable polarization is far easier to maintain over the duration of the writing process, which in these experiments varied from 1 to 20 h. One application leveraging these advantages is diffractive color, and we have demonstrated that subdividing the SLM into proportional diffracting areas can be used to print gratings that diffract R, G, and B combinations along a common +1 mode direction. This method of color definition using relative diffracting areas is uniquely advantageous given the programmability of independent diffracting areas within the SLM frame.

In closing, it is reasonable to foresee the continued evolution of this azopolymer-digital polarization optical printing platform toward practical diffractive optics applications and quite separately in applications requiring spatially tunable surface properties. While our overview emphasized the modularity and polarization-response of supramolecular azopolymers, this is mainly because they are straightforward to fabricate from commercially available starting materials and are easily transformed to stable microscale structures for use as static diffractive optics. Other applications emphasizing for example shape-shifting or dynamically reconfigurable surfaces might leverage other, covalently functionalized azopolymer systems. Regardless, the SLM-based digital polarization optical system is a material-independent approach. When coupled with evolving computational models linking the azopolymer molecular-architectural properties to the laser-written surface structure, it will likely form the basis of a highly versatile and experimentally accessible 2.5D microprinting platform.

## Supplementary Material



## Notes

Video showing the Mona Lisa micrograting array of Figure 14 as it is rotated with the camera in a fixed location (MPG)
